# PKC-Theta is a Novel SC35 Splicing Factor Regulator in Response to T Cell Activation

**DOI:** 10.3389/fimmu.2015.00562

**Published:** 2015-11-05

**Authors:** Robert Duncan McCuaig, Jennifer Dunn, Jasmine Li, Antonia Masch, Tobias Knaute, Mike Schutkowski, Johannes Zerweck, Sudha Rao

**Affiliations:** ^1^Discipline of Biomedical Sciences, Faculty of Education, Science, Technology and Maths, University of Canberra, Canberra, ACT, Australia; ^2^Department of Microbiology and Immunology, The Doherty Institute for Infection and Immunity, University of Melbourne, Melbourne, VIC, Australia; ^3^Department of Enzymology, Institute of Biochemistry and Biotechnology, Martin-Luther-University, Halle, Germany; ^4^JPT Peptide Technologies GmbH, Berlin, Germany

**Keywords:** SC35, PKC-theta, alternative splicing, T cells, histone marks, nuclear speckles

## Abstract

Alternative splicing of nuclear pre-mRNA is essential for generating protein diversity and regulating gene expression. While many immunologically relevant genes undergo alternative splicing, the role of regulated splicing in T cell immune responses is largely unexplored, and the signaling pathways and splicing factors that regulate alternative splicing in T cells are poorly defined. Here, we show using a combination of Jurkat T cells, human primary T cells, and *ex vivo* naïve and effector virus-specific T cells isolated after influenza A virus infection that SC35 phosphorylation is induced in response to stimulatory signals. We show that SC35 colocalizes with RNA polymerase II in activated T cells and spatially overlaps with H3K27ac and H3K4me3, which mark transcriptionally active genes. Interestingly, SC35 remains coupled to the active histone marks in the absence of continuing stimulatory signals. We show for the first time that nuclear PKC-θ co-exists with SC35 in the context of the chromatin template and is a key regulator of SC35 in T cells, directly phosphorylating SC35 peptide residues at RNA recognition motif and RS domains. Collectively, our findings suggest that nuclear PKC-θ is a novel regulator of the key splicing factor SC35 in T cells.

## Introduction

Alternative splicing of nuclear pre-mRNA transcripts is an essential regulator of eukaryotic gene expression. Alternative splicing results in numerous functionally distinct protein isoforms from a single gene (1). Pre-mRNA splicing takes place within the spliceosome, a ribonucleoprotein complex enriched in pre-mRNA splicing machinery including small nuclear ribonucleoproteins (snRNPs), spliceosome subunits, non-snRNP splicing factors, and a plethora of unknown mRNA-regulating nuclear factors (2, 3). Upon target transcript binding at specific splice sites, spliceosomes catalyze the removal of non-coding introns and exon ligation to produce protein-coding mRNA. A number of mechanisms regulate alternative splicing of pre-mRNA, including exon skipping, intron retention, and the selective use of 3′ and 5′ splice sites (4). Alternative splicing is a key mechanism for generating protein diversity and regulating gene expression and, therefore, plays an important role in cell function and development.

While most research has focused on transcriptional regulation of immune responses, alternative splicing of pre-mRNA is an emerging theme in the regulation of T cell function (5, 6). Several T cell genes, such as *CD44* and *CD45*, undergo alternative splicing to produce distinct protein isoforms (7, 8). Furthermore, antigenic stimulation alters the pattern of alternative splicing to produce significant functional changes in protein expression (8, 9). Recent global studies of alternative splicing have identified a novel group of genes that undergo activation-induced alternative splicing in T cells. Many of these genes encode proteins that are important for T cell function, such as RNA-binding proteins and transcription factors (10, 11). However, although numerous immunologically relevant genes undergo alternative splicing, the role of alternative splicing in T cell memory remains largely unexplored.

SC35 (also known as SRSF2) is a well-characterized splicing factor that belongs to the serine/arginine-rich (SR) protein family, an important class of splicing regulators. SR proteins have a conserved structure characterized by one or two N-terminal RNA recognition motifs (RRMs) and a C-terminal arginine-serine-rich (RS) domain that mediate RNA recognition and protein–protein interactions within the spliceosome, respectively. SC35 is exclusively nuclear and, therefore, plays a key role in nuclear processes (12). In addition to its crucial function as an alternative splicing regulator, SC35 also participates in transcriptional elongation, RNA stability, mRNA transport, and translation (13, 14). Furthermore, human SC35 binds exonic splicing enhancers under splicing conditions (15).

SC35 is associated with alternative splicing in T cells. For example, SC35 regulates alternative splicing of the *CD45* membrane receptor (16) and the cell adhesion molecule *CD44* in T cells (17). Moreover, SC35 is aberrantly expressed in immune-related diseases, including SLE, leukemia, and HIV (18–20). SC35 alternative splicing also promotes the inclusion and accumulation of oncogenes, such as Ron and HPV16 (21, 22). Interestingly, SC35 dysregulation has been implicated in neurodegenerative diseases, suggesting that SC35 may mediate other memory processes, such as cognitive memory, in addition to immune responses (23). These studies collectively demonstrate SC35’s important role in regulating immune responses to infections, but its role in T cell memory has not been examined.

Serine/arginine-rich splicing factors are phosphoproteins and are regulated by serine phosphorylation in the RS domain (23, 24). Several protein kinases have been shown to phosphorylate SR proteins (25), but the specific kinases that regulate SC35 in T cells are unknown. Several members of the protein kinase C (PKC) family, an evolutionarily conserved signaling kinase family, have been shown to regulate alternative splicing in many cell types including T cells (8, 26). Furthermore, both the PKC-α and PKC-δ isoforms have been shown to early-activate SC35 in post-natal rat cardiac muscle cells (27, 28). In T cells, PKC-θ is a central biochemical regulator that is essential for effective immune responses (29, 30). We have shown that PKC-θ is a novel nuclear epigenetic enzyme as well as a cytoplasmic signaling kinase. Nuclear-anchored PKC-θ forms an active signaling complex that directly binds to the promoter regions of inducible immune-responsive genes to regulate human T cell transcription (31). Given that several PKC family members have been shown to regulate alternative splicing events in T cells and that PKC-θ plays a key role in T cell function, we hypothesize that PKC-θ regulates SC35 in T cells.

Using a combination of Jurkat T cells, human primary T cells, and *ex vivo* naïve and effector virus-specific T cells isolated after influenza A virus infection, we show that SC35 phosphorylation (SC35p) is induced in response to stimulatory signals. Specifically, SC35p colocalizes with RNA polymerase II activated T cells and closely associates with H3K27ac (an active enhancer mark) and H3K4me3 (a promoter mark), which mark transcriptionally active genes. Interestingly, SC35 remains coupled to the active histone marks in the absence of continuing stimulatory signals. We show for the first time that nuclear PKC-θ co-exists with SC35 in the context of the chromatin template and is a key regulator of SC35 in T cells, directly phosphorylating SC35 peptide residues at RRM and RS domains. Collectively, our findings suggest that nuclear PKC-θ is a novel regulator of the key splicing factor SC35 in T cells.

## Materials and Methods

### Jurkat T Cell Culture

The Jurkat stimulation model was used as previously described (32). The human Jurkat T cell line (Clone E6-1, ATCC^®^ TIB-152) was cultured in complete 10% fetal bovine serum (FBS) RPMI media (Gibco, Life Technologies, Carlsbad, CA, USA). Jurkat T cells were either not stimulated (NS) or activated (ST) for 2 h at 5 × 10^5^ cells/mL with 24 ng/mL phorbol 12-myristate 13-acetate (PMA; Sigma-Aldrich, St. Louis, MO, USA; P8139) and 1 μM calcium ionophore (I; Sigma-Aldrich, A23187). For the stimulation model, previously activated Jurkat T cells were washed five times with stimulus-free medium and re-cultured for 3 days (SW) and subsequently re-stimulated (RST). For inhibitor studies, cells were pre-treated with rottlerin (Calbiochem) for 1 h prior to activation (31, 33).

### PKC-θ and Plasmid Transfections

Two full-length PKC-θ gene sequence constructs were used to create two plasmids with active or inactive nuclear localization: wild-type PKC-θ (PKCθ WT) or a PKC-θ gene sequence in which the non-canonical NLS sequence was inactivated by mutation (PKCθ NLS) as previously (34). Briefly, these sequences were cloned into the pTracer-CMV vector in frame with a C-terminal HA tag. Jurkat T cells were transiently transfected with 15 μg of vector-only plasmid, HA-tagged wild-type PKC-θ, or cytoplasmic-restricted PKC-θ plasmid using the NEON Transfection System Kit (Invitrogen, Life Technologies; MPK5000). Cells were subsequently stimulated as per the Jurkat stimulation model described above and fixed 48 h later in 2% formaldehyde and centrifuged onto poly-l-lysine-coated coverslips.

### Immunofluorescence/Image Acquisition

Coverslips of T cells were permeabilized by incubation with 1% Triton X-100 for 20 min and probed with a mouse antibody to phospho-epitopes of human SC35p (Abcam, Cambridge, UK; ab11826) followed by a secondary goat antibody to mouse Alexa-Fluor 568 (Lifetech A-10037). To examine colocalization, T cells were probed with an antibody mix of primary mouse antibody to SC35 with rabbit-raised antibodies targeting either human RNA-Pol-II-ser2p (Abcam ab5095), H3K4me3 (Merck 07-473), H3K27ac (Abcam ab4729), or PKC-θ (Abcam AB63365) followed by visualization with a secondary goat antibody to mouse immunoglobulins conjugated to Alexa-Fluor 568 and secondary antibodies to rabbit immunoglobulins conjugated to Alexa-Fluor 488 (Lifetech A-11008), respectively.

Coverslips were subsequently mounted on glass microscope slides with ProLong Gold anti-fade reagent (Life Technologies). Confocal laser scanning microscopy was used to study SC35 localization as previously described (35). 0.5 μm-spaced images were obtained with a Nikon x60 oil immersion lens on a Nikon C1 plus confocal system using NIS-Elements AR 3.2 software. The final image was obtained by averaging four sequential images from the same section at high resolution.

### Microscopy Data Analysis

Digital confocal images of PKC-θ were analyzed using the Fiji-ImageJ software (36) to determine the nuclear to cytoplasmic fluorescence ratio (Fn/c) using the equation: Fn/c = (Fn − Fb)/(Fc − Fb), where Fn is nuclear fluorescence, Fc is cytoplasmic fluorescence, and Fb is background fluorescence. A minimum of *N* = 20 cells were analyzed for each sample set. SC35 nuclear speckles were analyzed using Fiji-ImageJ software, with the nucleus of each cell and total nuclear fluorescence computed by the software. The Mann–Whitney non-parametric test (GraphPad Prism, GraphPad Software, San Diego, CA, USA) was used to determine significant differences between datasets.

### Microscopy Colocalization Analysis

For visualization of colocalization of confocal images, different image channels were overlaid in the same *Z*-plane. Consequently, a green and red overlay gave rise to yellow hotspots where the two molecules of interest were present in the same pixel locations. Statistical analysis of colocalization was performed with an intensity correlation coefficient-based method using the Colo-2 Fiji-ImageJ plugin with automatic thresholding (36–38). Pearson’s colocalization coefficients (PCCs) were collected using the ROI manager to select at least 20 individual nuclei per sample set. PCC could range from 1 to −1, with 1 denoting complete positive correlation, −1 complete negative correlation, and 0 no correlation. Data were presented as mean ± SEM. Statistical analyses were performed with Student’s *t*-test for paired comparisons. Additionally the Plot-Profile feature of Fiji-ImageJ was used to record the fluorescence intensity of a pair of antibody targets along a line through selected SC35 speckles. For each dataset, three speckles were counted for three separate cells and plotted with the mean ± SEM.

### Immunoblot Analysis

Immunoblot analysis was performed using primary mouse antibody to human SC35p (as above, ab11826) and secondary HRP-conjugated goat-anti-mouse antibody on nuclear extracts isolated from either primary human CD4^+^ cells, which were untreated (mock) or treated with Life Technologies siRNA PKC-θ pool (Life Technologies ID s11122, s11123), PKC-θ siRNA (Santa Cruz SC-36252), or nuclear extracts from NS or ST Jurkat T cells either untreated or treated with rottlerin. Nuclear extracts of transfected Jurkat T cells were also probed for nuclear expression of HA-tagged PKC-θ WT or NLS mutant constructs using a primary rabbit antibody to HA (Sigma, H6908). Signals were detected with enhanced chemiluminescence reagents (Western Lightning ECL-Plus, Perkin-Elmer NEL104001) and film exposure. Band intensity signals were normalized to the total protein transferred to the blot detected using the Quantitative Novex Reversible Protein Stain (Thermo Fisher Scientific IB7710) and Image J analysis.

### Half-Way ChIP Assays

Half-ChIP assays were performed according to the manufacturer’s instructions (Upstate Biotechnology) and as previously described for Jurkat T cells (31). Fixation was performed as detailed, and fixed chromatin was sonicated with an Ultrasonic processor (Qsonica) under optimized conditions to produce average DNA fragments of ~500 bp. Prior to antibody addition, samples were pre-cleared with salmon sperm DNA-protein A-agarose, and the soluble chromatin fraction was incubated overnight at 4°C with a primary antibody to PKC-θ and Protein A magnetic beads. The beads were washed and incubated with immunoblot loading buffer containing beta-mercaptoethanol at 95°C and analyzed as above (Immunoblot analysis) with a primary SC35 antibody.

### Kinase Profiling on Peptide Microarrays

Active recombinant PKC-θ was provided to JPT Peptide Technologies (Berlin, Germany) for kinase profiling on peptide microarrays. Unmodified SC35 peptides were chemoselectively immobilized on glass slides and incubated with kinase solution in the presence of γ-^33^P-ATP prior to high-resolution phosphorimaging. Spot recognition software packages, GenepixPro 7.2 and ArrayPro 4.0, were used for data analysis. Peptide constructs that displayed a normalized mean signal equal to or greater than two SDs above the mean were considered likely positive for phosphorylation events. Excel, R, and Python were used to determine the statistical significance of sequences and phosphorylation events.

### Total RNA Isolation and Quantitative Real-Time PCR

Total RNA was extracted and reverse transcription qPCR was performed as previously described (39) using the Maxima First-Strand cDNA Synthesis Kit (Thermo Scientific, Waltham, MA, USA) and 1:20 dilutions of cDNA for the RT-qPCR reaction. Ct values were normalized to the housekeeping gene *GAPDH*. Data were expressed as fold changes in message relative to NS. The following human TaqMan primer sets were used: *SC35 Hs01923929_s1* (Life Technologies), *GAPDH* Hs99999905 (Life Technologies).

## Results

### SC35p Is Induced in *Ex Vivo*-Derived Effectors Compared to Naïve T Cells in Response to Viral Infection

**Figure 1:**
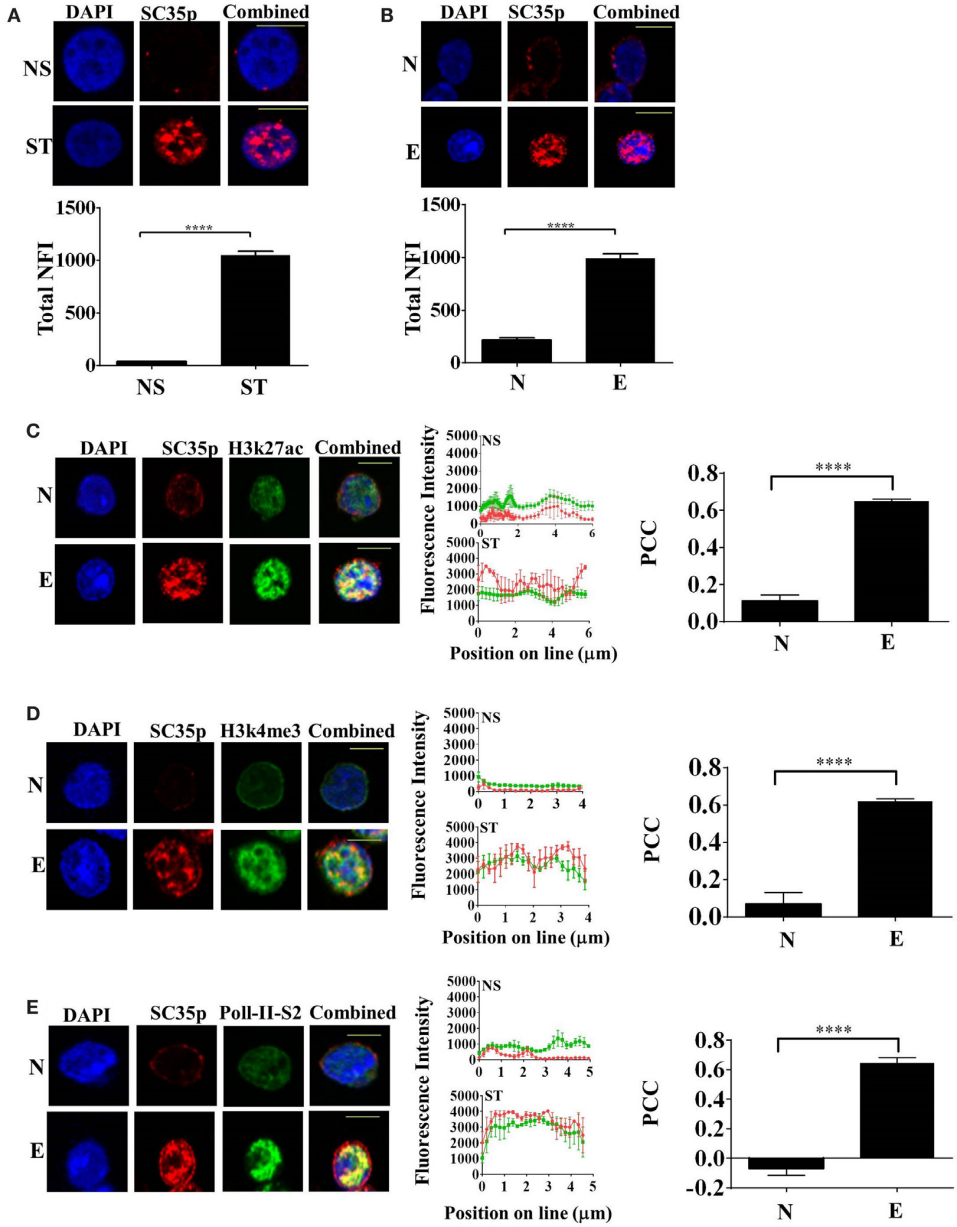
**Expression of phosphorylated SC35 in T cells and in primary mouse OT-1 T cells**. **(A)** Jurkat T cells were either unstimulated (NS) or PMA/I-activated (ST) for 2 h or **(B)** naive OT-1 CD8^+^CD44^lo/intermediate^ T-cells or influenza-specific effector OT-1 CD8^+^ T cells were fixed and probed with a mouse antibody to a phospho-epitope of SC35 followed by visualization with a secondary goat antibody to mouse immunoglobulins conjugated to Alexa-Fluor 568. Confocal laser scanning microscopy was used to measure SC35 expression as detailed in the section “Materials and Methods.” Representative images for each treatment are shown, with a 10-μm scale bar for **(A)** and a 5-μm scale bar for **(B)**. Total nuclear fluorescence intensity (NFI) was measured with Fiji-ImageJ. Data represent the mean ± SEM, *n* = 20 for each dataset with significant differences between datasets indicated. Naive OT-1 CD8^+^CD44^lo/intermediate^ T-cells or influenza-specific effector OT-1 CD8^+^ T cells were fixed and probed with a primary mouse antibody to a phospho-epitope of human SC35 and primary rabbit antibody to H3K27ac **(C)**, H3k4me3 **(D)**, or RNA-Pol-II ser-2 phosphorylation **(E)** followed by visualization with a secondary goat antibody to mouse immunoglobulins conjugated to Alexa-Fluor 568 and secondary antibodies to rabbit immunoglobulins conjugated to Alexa-Fluor 488, respectively. Confocal laser scanning microscopy was used to measure expression of SC35 and H3K27ac, H3k4me3, or RNA-Pol-II ser-2 as described in the section “Materials and Methods.” Representative images for each treatment are shown with a 5-μm scale bar. Channels were overlaid to examine colocalization of the antibody targets. Pearson’s colocalization coefficient (PCC) and mean fluorescent intensity line scans were calculated with Fiji-ImageJ as described in the section “Materials and Methods.” Data represent the mean ± SEM, *n* = 20 for each dataset with significant differences between datasets indicated. Red = SC35p; green = H3K27ac, H3k4me3, or RNA-Pol-II-ser2; and yellow = visual overlap between the fluorescence signals.

Given that the splicing factor SC35 regulates alternative splicing in T cells, we initially determined the subcellular distribution of SC35 by fluorescence microscopy in the human Jurkat T cell line; cells were either non-stimulated or stimulated with phorbol-myristate acetate and calcium ionophore (PMA/I), a known PKC pathway inducer. The phosphorylated epitope of SC35 (SC35p) was detected. Immunofluorescence analysis showed that the total nuclear fluorescence of SC35p was substantially increased following primary stimulation compared to non-stimulated Jurkat T cells (Figure 1A).

We next investigated SC35p dynamics in naïve and effector T cells utilizing an established virus infection model in which naive (CD44^lo^CD62L^hi^) OT-I TCR transgenic CD8^+^ T cells specific for the ovalbumin peptide (OVA257-264) were adoptively transferred into congenic C57BL/6J (B6) hosts followed by intranasal (i.n.) infection with the A/HKx31-OVA virus (40). Immunofluorescence analysis of sort-purified (>99% purity, Figures S1A,B in Supplementary Material) naive (day 0) and effector (day 10) T cells was performed. Consistent with the activated Jurkat T cells, SC35p was markedly increased in virus-specific effector T cells compared to naïve T cells (Figure 1B). Overall, our findings suggest that SC35p is induced in a stimulus-dependent manner in both the Jurkat T cell line and virus-specific T cells.

### SC35p Spatially Overlaps with Active Histone Marks in Effector T Cells in Response to Viral Infection

Highly compacted chromatin structures enriched in nucleosomes are transcriptionally silent. Chromatin accessibility is pivotal in regulating gene expression and can be orchestrated via a number of mechanisms including the addition of PTMs to histone proteins (41–43). To determine if SC35p localizes to transcriptionally active genes during T cell differentiation in response to infection, we double-stained with SC35p and histone H3K27ac (Figure 1C), H3K4me3 (Figure 1D), or RNA polymerase II-serine2 phosphorylation (Figure 1E), which are associated with transcriptionally active or poised genes (44, 45) in naïve (day 0) and effector (day 10) OT1 CTLs. SC35p was strongly colocalized with all three active marks after differentiation into effector CTLs: PCC 0.65 ± 0.01 H3k27ac (**C**); PCC 0.62 ± 0.01 H3k4me3 (**D**); PCC 0.64 ± 0.03 RNA polymerase II-serine2 (**E**). These findings suggest that SC35p co-exists within key regulatory regions strongly linked with active enhancers (H3K27ac) and gene promoter regions (H3K4me3) in the context of virus-specific CTL differentiation.

### SC35p Remains Localized to Active Histone Marks in the Absence of Continuous T Cell Activation Signals

**Figure 2:**
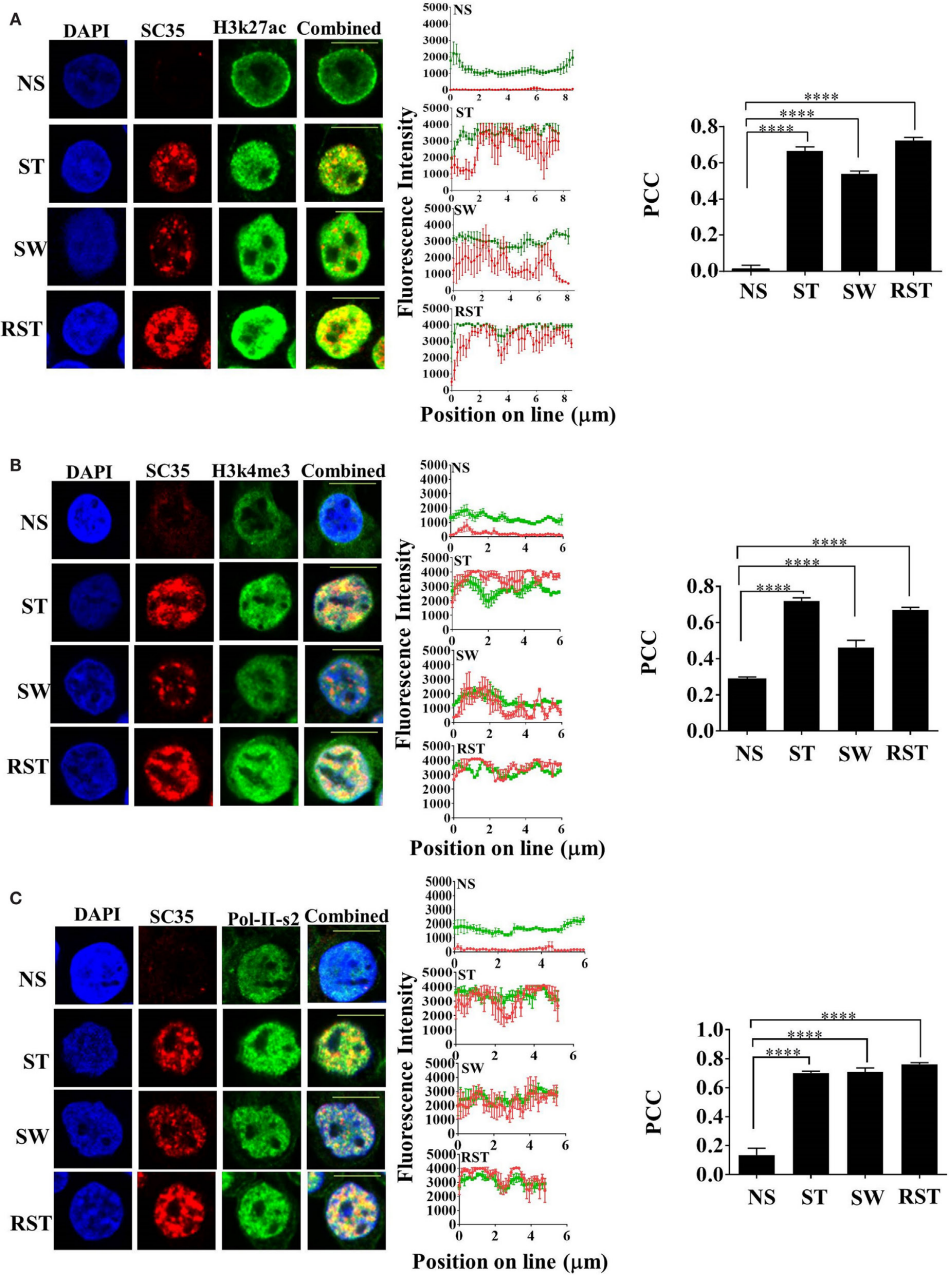
**Interplay between SC35p, histone PTMs, and RNA-Pol-II ser2 in the Jurkat T cell model**. Jurkat T cells stimulated as described in the section “Materials and Methods” (NS, no stimulation; ST, stimulation; SW, stimulus withdrawal; RST, re-stimulation) were fixed and probed with a primary mouse antibody to a phospho-epitope of human SC35 and primary rabbit antibody to H3K27ac **(A)**, H3k4me3 **(B)**, or RNA-Pol-II ser-2 **(C)** followed by visualization with a secondary goat antibody to mouse immunoglobulins conjugated to Alexa-Fluor 568 and secondary antibodies to rabbit immunoglobulins conjugated to Alexa-Fluor 488, respectively. Confocal laser scanning microscopy was used to measure expression of SC35p and H3K27ac, H3k4me3, or RNA-Pol-II-ser-2. Representative images for each stimulation point are shown with a 10-μm scale bar. Channels were overlaid to examine colocalization of the antibody targets. Pearson’s co-localizaton coefficient (PCC) and mean fluorescent intensity line scans were calculated with Fiji-ImageJ as described in the section “Materials and Methods.” Data represent the mean ± SEM, *n* = 20 for each dataset with significant differences between datasets indicated. Red = SC35; green = H3K27ac, H3k4me3, or RNA-Pol-II-ser2; and yellow = visual overlap between the fluorescence signals.

Nuclear accumulation of SC35p correlates with active transcription. To assess the time-dependency of nuclear SC35p after continuous T cell activation, an experiment was designed (see schematic, Figure S2A in Supplementary Material) to measure SC35p and its ability to colocalize with active PTMs before (NS), during (ST), and after (cells were washed five times to remove stimulus and maintained for three divisions; SW) T cell activation or after RST. Double staining with the active transcription marks H3K27ac (Figure 2A), H3K4me3 (Figure 2B), and RNA Pol II (Figure 2C) indicated that SC35p strongly colocalized with all three histone marks in both primary stimulation (ST) (Figures 2A–C; PCC 0.66 ± 0.02 H3k27ac; PCC 0.72 ± 0.017 H3k4me3; PCC 0.7 ± 0.01 RNA Pol II-ser2) and secondary (RST) stimulation conditions (Figures 2A–C; PCC 0.72 ± 0.019 H3k27ac; PCC 0.67 ± 0.01 H3k4me3; PCC 0.76 ± 0.01 RNA Pol II-ser2). Interestingly, this favorable colocalization was also observed in the absence of ongoing stimulation in SW Jurkat cells (Figures 2A–C PCC 0.0.54 ± 0.016 H3k27ac; PCC 0.46 ± 0.03 H3k4me3; PCC 0.71 ± 0.02 RNA Pol II-ser2). In contrast, no colocalization of these active transcription marks was observed in NS Jurkat T cells. Thus, SC35p is induced following primary activation and is maintained with active chromatin marks after stimulus withdrawal and further induced following secondary stimulation. It should be noted that *SC35* gene expression was unchanged under all conditions (Figures S3A,B in Supplementary Material). These data suggest that primary stimulation is required for induction of SC35 phosphorylation and for its co-existence with active chromatin marks and this association with chromatin is maintained in the absence of continued activation signals.

### PKC-θ Regulates SC35p in Activated T Cells

**Figure 3:**
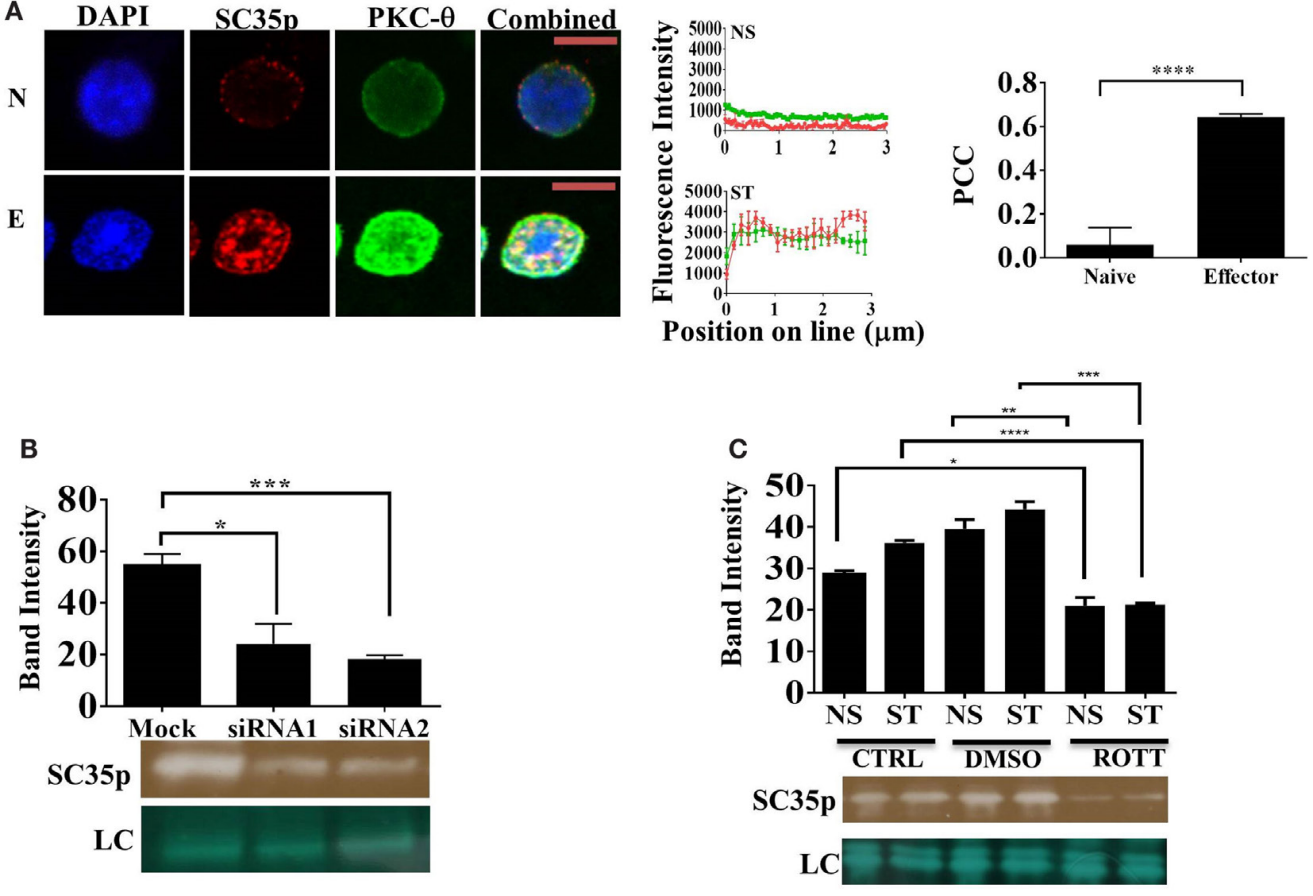
**Interplay between PKC-**θ** and SC35 phosphorylation**. **(A)** Naive OT-1 CD8^+^CD44^lo/intermediate^ T-cells or influenza-specific effector OT-1 CD8^+^ T-cells were fixed and probed with a primary mouse antibody to a phospho-epitope of SC35 and primary rabbit antibody to PKC-θ followed by visualization with a secondary goat antibody to mouse immunoglobulins conjugated to Alexa-Fluor 568 and secondary antibodies to rabbit immunoglobulins conjugated to Alexa-Fluor 488, respectively. Confocal laser scanning microscopy was used to measure expression of SC35 and PKC-θ. Representative images for each stimulation point are shown with a 5-μm scale bar. Channels were overlaid to examine colocalization of the antibody targets. Pearson’s colocalization coefficient (PCC) and mean fluorescent intensity line scans were calculated with Fiji-ImageJ as described in the section “Materials and Methods.” Data represent the mean ± SEM, *n* = 20 for each dataset with significant differences between datasets indicated. Red = SC35; green = PKC-θ; and yellow = visual overlap between the fluorescence signals. **(B)** Cell lysates of primary human CD4^+^ cells were untreated (mock) or treated with PKC-θ siRNA1 (Life Technologies) or siRNA2 (Santa Cruz). Effect on SC35 was analyzed by immunoblotting with a mouse raised primary antibody to a phospho-epitope of SC35, measuring band intensity with Fiji-ImageJ for each sample. A representative image of SC35 labeling for three separate experiments (*n* = 3) is displayed (labeled SC35), with the mean intensity plotted with significant differences displayed for each treatment along with a representative loading control (LC) as described in the section “Materials and Methods.” The effect of rottlerin treatment **(C)**, a PKC-θ-specific kinase inhibitor, on SC35 phosphorylation was also examined by immunoblotting as described above. A representative loading control (LC) is shown along with a representative SC35p-probed blot for three separate experiments (*n* = 3). The mean intensity is plotted with significant differences displayed for each treatment.

Several protein kinases capable of phosphorylating SR proteins have been described (23, 24, 27, 28). However, the specific SC35-regulating kinases in T cells have yet to be identified. Given that PKC-θ is a critical regulator of gene expression in human T cells (29, 30), we speculated that PKC-θ might also regulate SC35 expression in Jurkat T cells. Double staining and confocal microscopy revealed strong colocalization of PKC-θ and SC35p in viral influenza-specific OT-1 CD8^+^ effector T cells (Figure 3A; PCC 0.64 ± 0.013). In contrast, no colocalization was detected in naïve OT-1 T cells (Figure 3A, PCC 0.06 ± 0.07). Next, we investigated the impact of PKC-θ knockdown on SC35p by immunoblotting with anti-SC35p in either mock or PKC-θ siRNA1 (Lifetech) and siRNA2 (Santa Cruz)-treated nuclear extracts of primary human bulk CD4^+^ T cells. Both siRNA pools substantially inhibited SC35 phosphorylation relative to the mock treatment (Figure 3B).

Next, we assessed the impact of PKC-θ catalytic activity on SC35p. PKC-θ catalytic activity is necessary for chromatin association and inducible gene expression (31). To assess the impact of PKC-θ catalytic activity, we used an ATP-competitive PKC-θ inhibitor, which has previously shown to inhibit PKC-θ activity in human T cells (31, 33). Immunoblotting for SC35p in rottlerin or vehicle pre-treated NS and ST Jurkat T cell nuclear extracts displayed a significant reduction in SC35p phosphorylation compared to the mock-treated cells. Collectively, these data indicate a role for PKC-θ kinase activity in the active phosphorylation of SC35 in T cells (Figure 3C).

### Nuclear PKC-Theta and SC35p Co-exist in Jurkat T Cells

**Figure 4:**
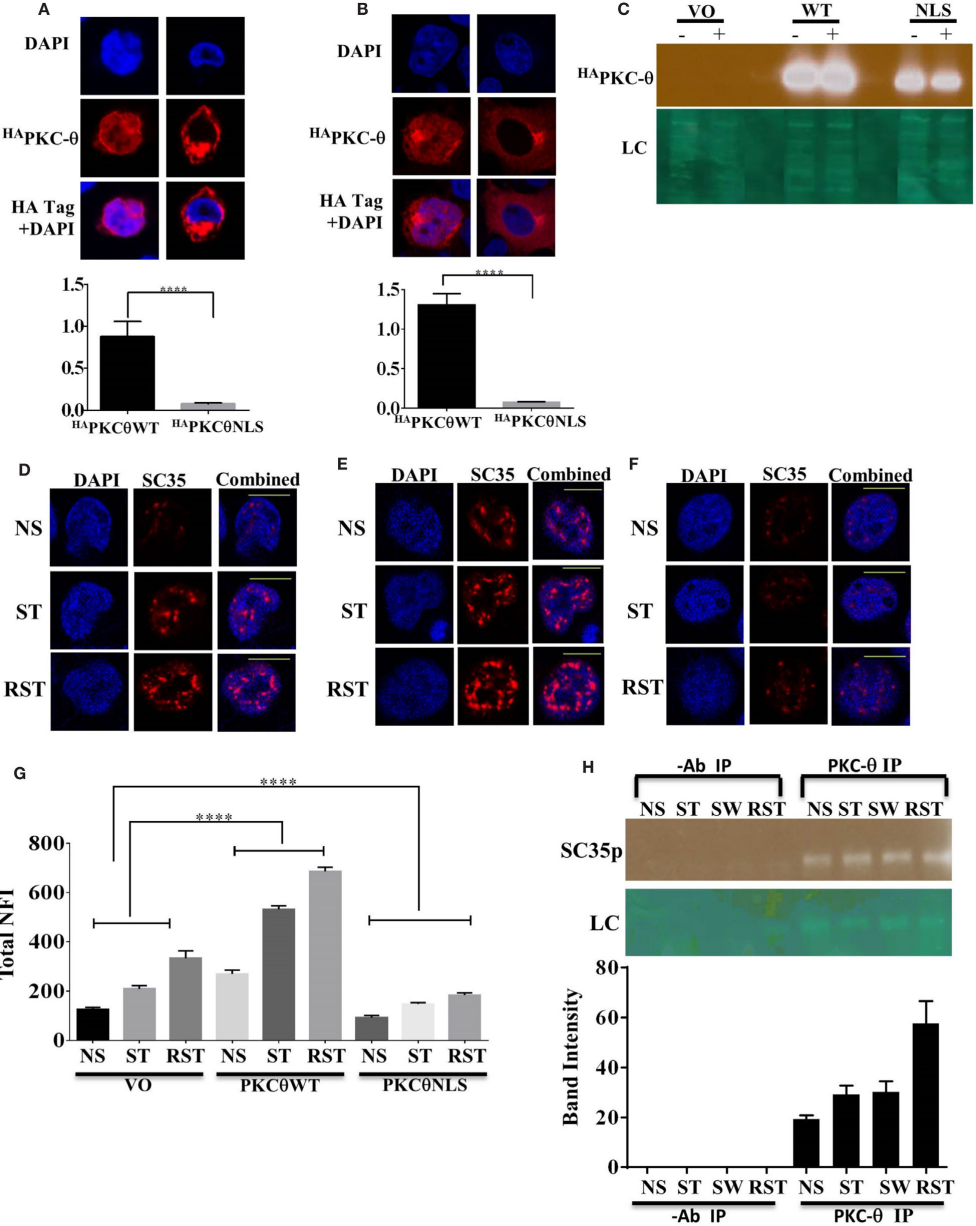
**Localization of PKC-**θ** in T cells and effect on SC35p**. The full-length PKC-θ wild-type gene sequence (^HA^PKC-θ WT) and the NLS mutation (^HA^PKC-θ NLS) were cloned into the pTracer-CMV vector in frame with a C-terminal HA tag. Transfected Hut-78 T cells **(A)** and Jurkat T cells **(B)** and were fixed and probed with a rabbit antibody to the HA-tag followed by visualization with a secondary goat antibody to rabbit immunoglobulins conjugated to Alexa-Fluor 568. Confocal laser scanning microscopy was also used to assess PKC-θ localization as described in the section “Materials and Methods.” Representative images for each construct are shown. Fn/c values for each construct are shown with significant differences between datasets indicated. Data represent the mean ± SEM, *n* = 20 for each dataset. **(C)** Jurkat T cells were transfected, stimulated, and nuclear extracts created to examine nuclear localization of the PKC-θ wild-type and PKCθNLS mutant. A representative immunoblot image of nuclear extracts showing ^HA^PKC-θ localization in the nucleus is shown. Jurkat T cells were transfected as described in the section “Materials and Methods” and subsequently stimulated (NS, non-stimulated; ST, stimulated; RST, re-stimulated). Cells were fixed and probed with a mouse antibody to a phospho-epitope of human SC35 with secondary antibodies to mouse immunoglobulins conjugated to Alexa-Fluor 568. Confocal laser scanning microscopy was used to study SC35p expression in HA tag-positive cells. Representative images for each construct are shown with a 10-μm scale bar: **(D)** vector only (VO), **(E)**^HA^PKC-θ WT, and **(F)**^HA^PKC-θ NLS. Total nuclear fluorescence values for each construct are shown with significant differences between datasets indicated **(G)**. Data represent the mean ± SEM, *n* = 20 for each dataset. **(H)** Nuclear extracts were made from Jurkat T cells stimulated as previously described (NS, no stimulation; ST, stimulation; SW, stimulus withdrawal; RST, re-stimulation) and subjected to half-way CHIP using PKC-θ pull down or a no antibody control. Samples were probed with a primary mouse antibody to a phospho-epitope of human SC35 as described in the section “Materials and Methods”; representative bands are shown. SC35 band intensity was plotted using Fiji-ImageJ software minus background for *n* = 3 with mean ± SEM.

We have previously shown that PKC-θ can function as a nuclear epigenetic enzyme in T cells as well as a cytoplasmic signaling kinase (31). To distinguish the cytoplasmic and nuclear roles of PKC-θ in SC35p expression, previously validated PKC-θ constructs (34), a HA tagged wild-type PKC-θ (^HA^PKC-θ WT) or a HA tagged PKC-θ with a mutated NLS (^HA^PKC-θ NLS), were expressed in Jurkat T cells. Consistent with our previous observations, ^HA^PKC-θ NLS was significantly cytoplasmic restricted compared to the ^HA^PKC-θ WT in the Jurkat and HUT T cell lines (Figures 4A–C). Next, to determine the effect of nuclear PKC-θ regulation on SC35 phosphorylation in Jurkat T cells, the subcellular distribution of SC35p was analyzed in Jurkat T cells transfected with vector-only (mock) control and PKC-θ plasmid constructs and then stimulated 48 h after transfection in the Jurkat stimulation model. Total nuclear SC35p fluorescence significantly increased in all ^HA^PKC-θ WT-treated cells (Figure 4E) overexpressing PKC-θ compared to mock constructs (Figure 4D), suggesting that PKC-θ plays a role in speckle formation. In contrast, transfection with the ^HA^PKC-θ NLS mutant resulted in diminished SC35p in the Jurkat T cells (Figure 4F), as indicated in the nuclear fluorescence data (Figure 4G).

To determine whether nuclear PKC-θ and SC35 co-exist in the nucleus in the context of the chromatin template, we performed PKC-θ half-way ChIP on Jurkat T cell nuclear extracts. Endogenous PKC-θ and SC35p were associated in Jurkat T cells, particularly in RST Jurkat T cells (Figure 4H). Interestingly, co-existence of both proteins was maintained in SW cells following stimulus withdrawal (Figure 4H), but this association increased following secondary stimulation (Figure 4H). Taken together, PKC-θ and SC35p co-exist in the proximity of chromatin within the nuclear compartment of T cells.

### PKC-θ Directly Phosphorylates SC35 at Regulatory Domains

To determine whether PKC-θ directly phosphorylates SC35 residues, an *in vitro* kinase assay was performed in which SC35 peptide constructs were incubated with active PKC-θ in the presence of radiolabeled γ^33^P-ATP in an array-based format.

**Figure 5:**
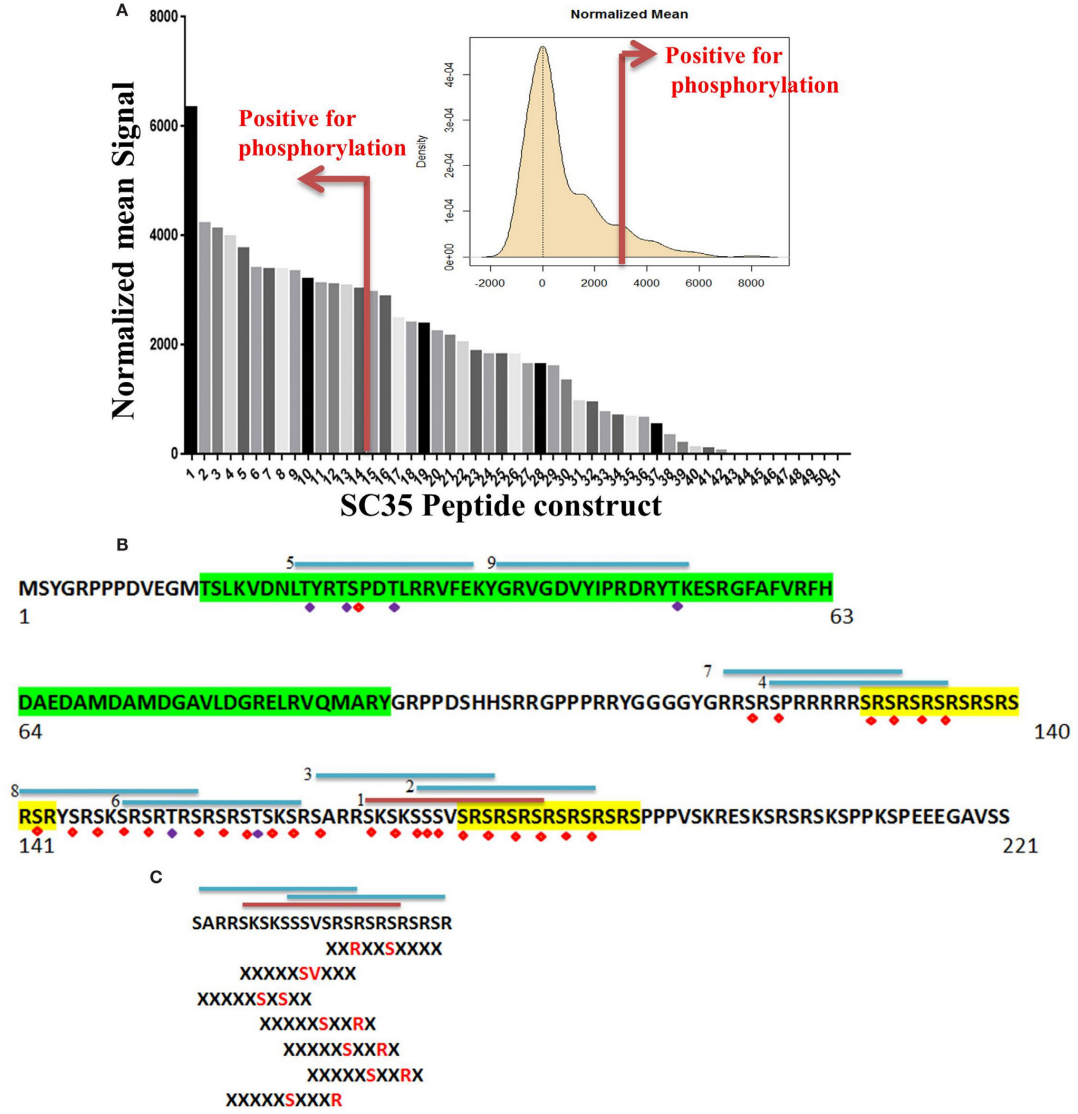
**Identification of phosphorylated residues on the SC35 splicing protein. (A)** Mean peptide signal and all SC35 peptide constructs used to examine SC35 for phosphorylation by PKC-θ. The red bar indicates the cut-off for positive signal for phosphorylation. The distribution curve for SC35 peptide phosphorylation is also displayed: the *y*-axis is the kernel density estimate of the normalized phosphorylation signal intensities on the *x*-axis, with anything equal to or greater than the red bar (2 × SD above the mean) considered a positive phosphorylation event. **(B)** The amino acid sequence of SC35 indicating the location of the top nine peptides in descending order and their location; the other five peptides correspond to overlaps with the first nine peptide locations. Green highlight denotes the RRM domain, the yellow highlight denotes RS dipeptide repeats. Peptides are numbered in order of mean signal intensity: red bar is the highest signal, blue bars are the other positive SC35 peptides, and possible serine or threonine phosphorylation residues are indicated by red or blue diamonds. **(C)** The top three peptide sequences and their overlap with the SC35 amino acid sequence. Hutti et al. (46) reported a serine/threonine specific motif for PKC-θ-mediated phosphorylation; using this information, the section of SC35 that scored the top three hits for peptide phosphorylation signals was analyzed for these motifs. Seven separate phosphorylation motifs were identified.

Of the 51 peptide constructs tested, 14 were positive for phosphorylation events (Figure 5A). SC35 contains two long stretches of RS repeats and an RRM domain (12). Sequence analysis of these 14 peptides showed that all but two of the positive peptides localized to the long tract dipeptide RS domains (Table 1). In contrast, peptides 5 and 9 localized to the RRM domain at the N-terminal. The peptide with the strongest phosphorylation signal (peptide 1) was located in the RS domain (171AA–186AA; Figure 5B).

**Table 1:** **Mean peptide signals and protein domains, related to Figure 6**.

	SC35 peptide sequence	Mean	Normalized mean	SC35 protein domain
Peptide 1	SKSKSSSVSRSRSRS	55,706	6,331	RS domain
Peptide 2	SSSVSRSRSRSRSRS	53,589	4,214	RS domain
Peptide 3	SARRSKSKSSSVSRS	53,504	4,129	RS domain
Peptide 4	SPRRRRRSRSRSRSR	53,345	3,970	RS domain
Peptide 5	LTYRTSPDTLRRVFE	53,132	3,757	RRM domain
Peptide 6	SRSRTRSRSRSTSKS	52,781	3,406	RS domain
Peptide 7	RRSRSPRRRRRSRSR	52,746	3,370	RS domain
Peptide 8	RSRYSRSKSRSRTRS	52,745	3,370	RS domain
Peptide 9	YGRVGDVYIPRDRYT	52,722	3,346	RRM domain
Peptide 10	PPVSKRESKSRSRSK	52,575	3,199	RS domain
Peptide 11	SRSKSRSRTRSRSRS	52,497	3,121	RS domain
Peptide 12	SRSTSKSRSARRSKS	52,475	3,100	RS domain
Peptide 13	SKSRSARRSKSKSSS	52,461	3,086	RS domain
Peptide 14	SRSRSRSRSRSPPPV	52,402	3,026	RS domain

*The mean peptide signals for all 14 SC35 peptide constructs above the positive cut-off for phosphorylation. The peptide sequence and the protein domain occupied are also tabulated*.

We also examined the relationship between phosphorylated peptides and recently identified PKC-θ motifs (Figure 5C) (46). The strongest peptide signals (peptides 1, 2, and 3) contained seven PKC-θ phosphorylation motifs. Furthermore, the other 11 phosphorylation-positive peptides also contained regions that correlated with putative PKC-θ phosphorylation motifs. Overall, our data demonstrate that the nuclear PKC-θ kinase directly phosphorylates SC35 at both RMM and RS regulatory domains.

## Discussion

The contribution of alternative splicing to T cells is an emerging area of immunological research. The interplay between the transcriptional splicing machinery and the chromatin landscape is poorly defined, particularly in T cells. Here, we show that the key splicing factor SC35 is induced in response to T cell stimulatory signals. Specifically, we show that phosphorylated form of SC35 (SC35p) is enriched following T cell activation in Jurkat T cells, human primary T cells, and *ex vivo* effector virus-specific T cells isolated after influenza A virus infection. We show that SC35p colocalizes with RNA polymerase II in activated T cells and spatially overlaps with H3K27ac and H3K4me3, which mark transcriptionally active genes in primary and secondary T cell activation. PKC-θ is a novel regulator of SC35 expression in T cells that directly phosphorylates SC35 at its key regulatory regions.

Several PKC family members play a role in mRNA splicing in other cell types (8, 26–28). Here, we show that PKC-θ is a key regulator of SC35 in human T cells, and for the first time demonstrate that nuclear PKC-θ regulates SC35 phosphorylation in T cells. The increase in SC35 phosphorylation in PKC-θ WT-transfected T cells suggests that phosphorylation by PKC-θ initiates speckle formation and an increase in SC35p signal and concentration. Interestingly, DAG kinase has also been shown to localize in the nucleus (47), and it would be interesting to know whether it is located in a similar active transcription complex as PKC-θ in T cells. In addition, PIPKs, part of a PKC co-factor pathway, associate with nuclear speckles, further suggesting that PKCs and PKC co-factors play a role in splicing (48).

We demonstrate that PKC-θ directly phosphorylates SC35 at RS and RRM domains. Furthermore, our data with a PKC catalytic inhibitor and siRNA knockdown suggests that PKC-θ is critical for SC35 phosphorylation in T cells. The RS domain mediates protein–protein interactions, such as subcellular localization (49), nuclear export, and retention signals (12), and functions as a splicing activator (50). We also identified two PKC-θ-phosphorylated peptides that lie in the RRM domain. The RRM domain recognizes RNA recognition sequencing and has been shown to mediate subcellular localization (49) and alternative splicing specificity (51, 52). Future studies will be required to address the essential nature of these novel PKC-θ-targeted phosphorylation sites within key SC35 domains in T cells. It has been reported that Tip60 acetylation of SC35 at lysine residue 52 promotes proteasomal degradation of splicing factors and reduces SC35 phosphorylation (53). Given that Lysine 52 is in proximity to putative PKC-θ phosphorylation sites in the RRM domain, and siRNA and rottlerin inhibitor experiments implicate PKC-θ kinase activity in SC35 RRM domain phosphorylation, an intriguing possibility exists that this phosphorylation by PKC-θ may play a role in preserving the SC35 nuclear speckle from degradation in T cells.

Memory T cells elicit fast and enhanced secondary immune responses upon antigen exposure to mount effective adaptive immune responses to infectious diseases and cancers (54–56). Enhanced memory T cell function is underpinned by the rapid induction of a distinct cohort of genes in response to antigenic re-challenge (57–59). This transcriptional response is greater and more robust in memory than naïve T cells. Furthermore, memory T cells “remember” previous transcriptional responses for decades in humans in the absence of antigen (60). Although the rapid recall responses of memory T cells are well documented, the signaling pathways and mechanisms that regulate T cell transcriptional memory are complex and have yet to be fully elucidated (61–63). We show in this study that SC35p colocalizes with RNA Pol II and key histone marks in primary and secondary-activated T cells. An interesting finding was that SC35p remained closely associated with key histone marks in SW Jurkat T cells in spite of the absence of activating signals. Furthermore, SC35p and PKC-θ co-existed in close proximity to the chromatin platform following stimulus withdrawal. The maintenance of SC35 in SW Jurkat T cells suggests that SC35 exists in a poised or ready state following stimulus withdrawal. This poised state may serve as a transcriptional memory signature that marks genes for rapid transcription upon secondary activation. We show that SC35 colocalizes with active histone marks H3K27ac and H3K4me3; however, while H3K27ac marks active enhancers, H3K4me3 is largely deposited at promoter regions (45, 64). Given that H3K4me3 is increased in memory T cells and in effector cells at active gene loci (65), the role of SC35 at gene enhancers in memory T cells deserves further study. Since SC35 is also implicated in chromatin remodeling (66), proteomic analysis will be required to explore the interplay between the enzymes that regulate T cell memory and SC35 in T cells.

**Figure 6:**
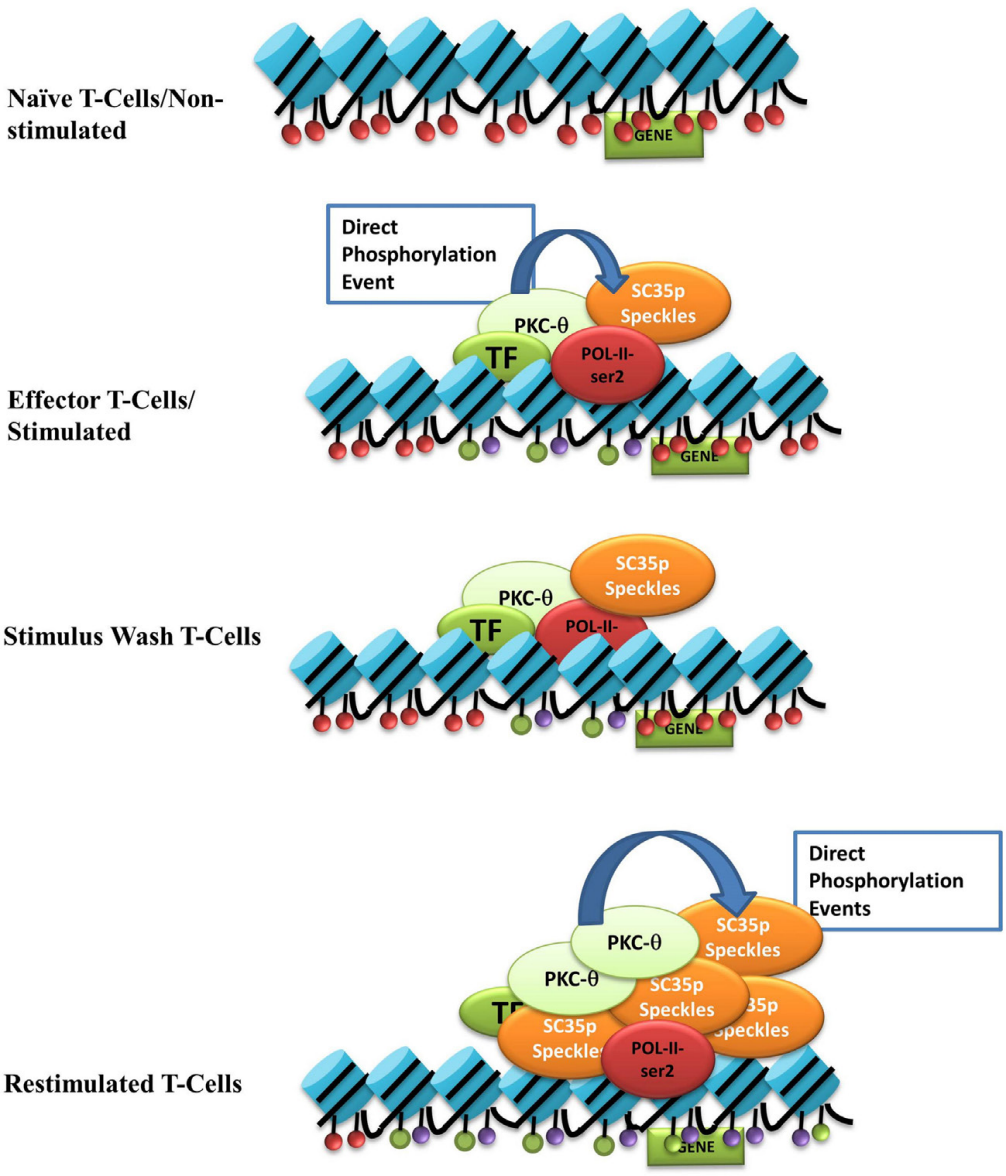
**Nuclear PKC-**θ** as a novel regulator of SC35 in T-cells**. Our model of the interaction between PKC-θ and SC35 in the context of TM in Jurkat T cells. Blue cylinders represent histones, and the colored ovals represent histone PTMs: red = repressive, purple (H3k27ac), or green (H3k4me3). Light green oval is PKC-θ, red oval is RNA-Pol-II, the darker green oval represents various TFs, and the orange oval is SC35p speckles. Upon stimulation, PKC-θ enters the nucleus and binds to a chromatin platform incorporating RNA-Pol-II and various transcription factors. At the same time, PKC-θ phosphorylates SC35, relocating the speckle to the site of transcription to potentially form a linked complex between the chromatin platform, the splicing speckle, and active histone marks. After stimulus withdrawal, this association between PKC-θ/TFs, histone PTMs, and SC35 speckles is maintained and expands even further upon re-stimulation to effect massive recruitment of phosphorylated SC35 splicing speckles.

We propose a model in which nuclear PKC-θ interacts with SC35 in distinct T cell activation states (see Figure 6). Upon stimulation, nuclear PKC-θ forms an active transcription complex with RNA Pol II as previously described (31). At the same time, PKC-θ directly phosphorylates SC35 speckles at RS and RRM domains to direct localization of SC35p near active gene expression regions, such as H3K27ac-marked enhancers, and initiates speckle formation. Following stimulus removal, the SC35p speckle and PKC-θ complex are maintained in a poised or ready state near accessible chromatin. Reactivation signals increase SC35p and further induce phosphorylation of SC35p speckles. Together, future studies will be required to provide in-depth mechanistic insights into the contribution of SC35p in marking genes for rapid reactivation.

Given that PKC-θ regulates the key splicing factor SC35, future studies should aim to address the role of PKC-θ in alternative splicing utilizing global, high-throughput methods, such as RNA sequencing. It will also be important to determine if nuclear kinase-regulated splicing is unique to T cells or whether other kinases, such as Akt and CDK5/p35, which mediate CD8^+^ T cell differentiation and neuronal development, play a nuclear role in splicing. Unraveling the role of alternative splicing in T cell regulation is likely to be useful in translational applications in autoimmune diseases and cancer biology.

## Author Contributions

RM conducted the experiments for Figures 1–4, carried out the analysis, and wrote parts of the paper; JD conducted the experiment for Figure 3C with RM and wrote the paper with SR; JL prepared virus-specific T cells; AM, TK, MS, and JZ conducted the experiments for Figure 5. SR: proposed the concept, helped with analysis, and wrote the paper.

## Conflict of Interest Statement

The authors declare that the research was conducted in the absence of any commercial or financial relationships that could be construed as a potential conflict of interest.
