# Supplementary figures and images for: PKC-Theta is a Novel SC35 Splicing Factor Regulator in Response to T Cell Activation
Source: Front Immunol. 2015 Nov 5;6:562. doi: 10.3389/fimmu.2015.00562 (PMC4633479; doi:10.3389/fimmu.2015.00562)

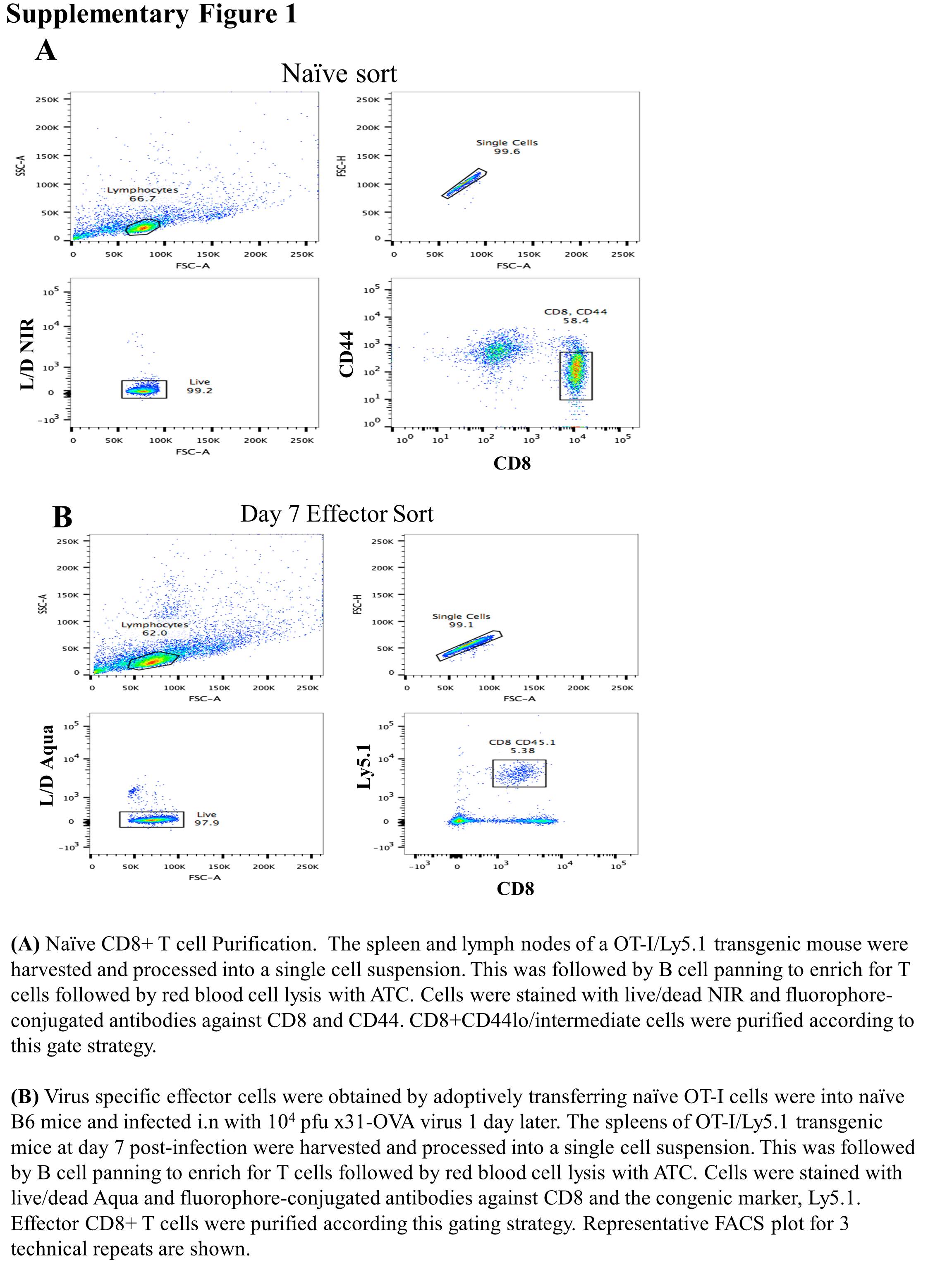

Supplement: Supplementary file 1 [file image_1.jpeg]

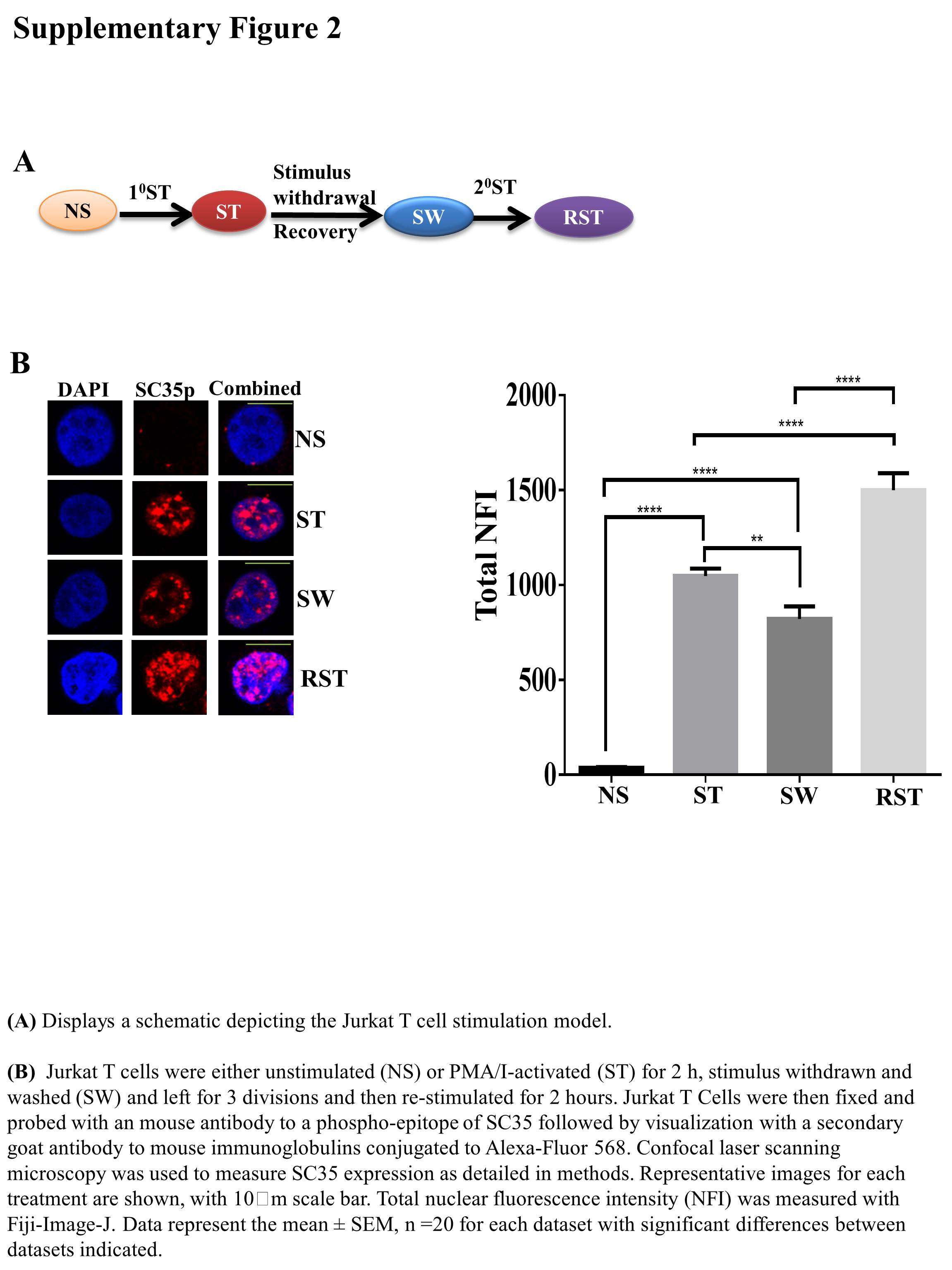

Supplement: Supplementary file 2 [file image_2.jpeg]

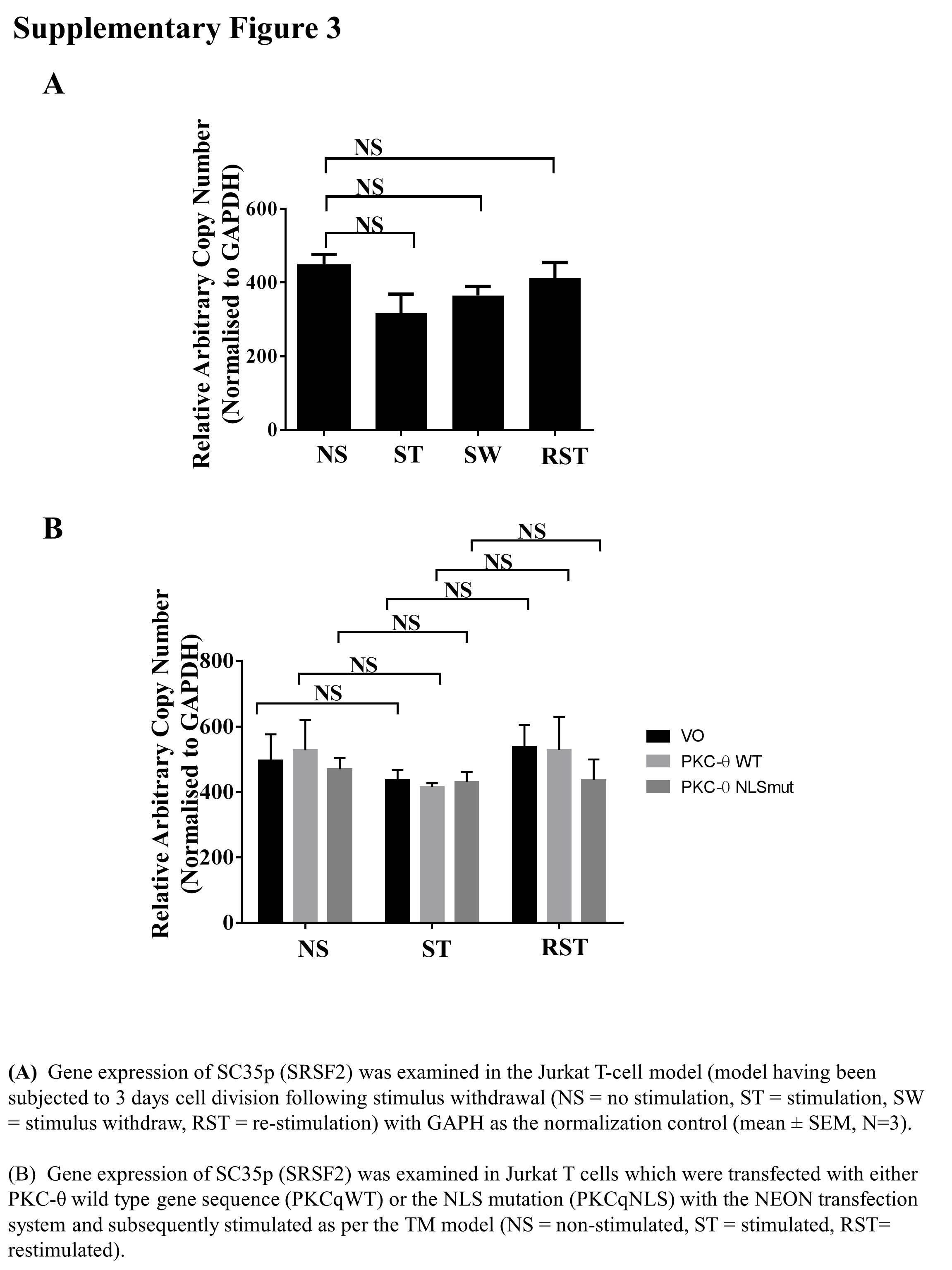

Supplement: Supplementary file 3 [file image_3.jpeg]
